# Supplementary material for: Machine Learning Identifies Key Proteins in Primary Sclerosing Cholangitis Progression and Links High CCL24 to Cirrhosis
Source: Int J Mol Sci. 2024 May 30;25(11):6042. doi: 10.3390/ijms25116042 (PMC11173115; doi:10.3390/ijms25116042)
Supplement: Supplementary file 1 [file ijms-25-06042-s001.zip › supplementary figures.pdf]

Supplementary materials

Supp. Figure S1

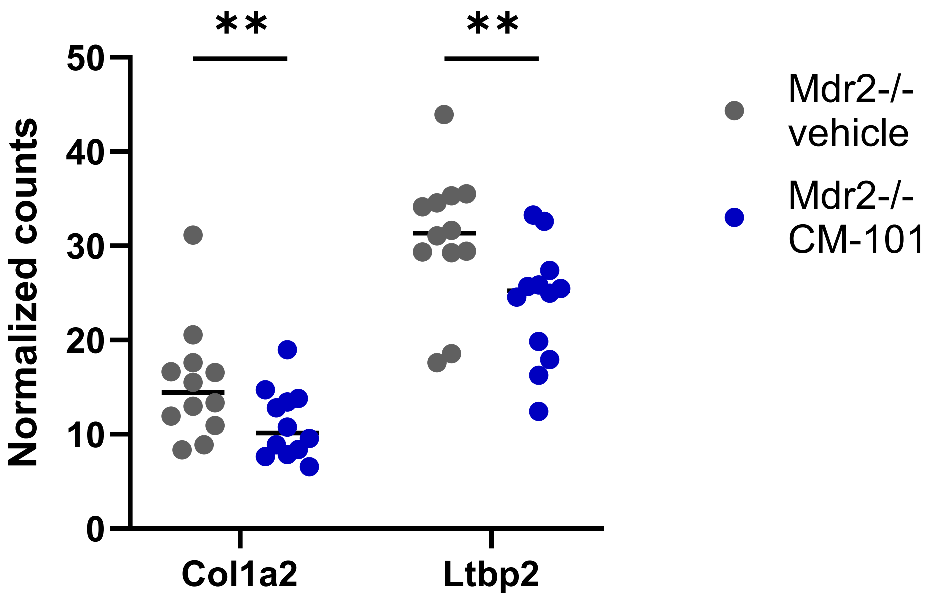


Reduction in ltbp2 after treatment with CM-101 in Mdr2-KO model mice. Paraffin-embedded liver sections were analyzed for mouse whole transcriptome atlas. Holm-Šídák's multiple comparisons test comparing the mean values of normalized counts for Col1a2 gene in Pan-cytokeratin-positive area, and for Ltbp2 gene in Pan-cytokeratin-negative area. **, $p<0.01$.

Supp. Figure S2

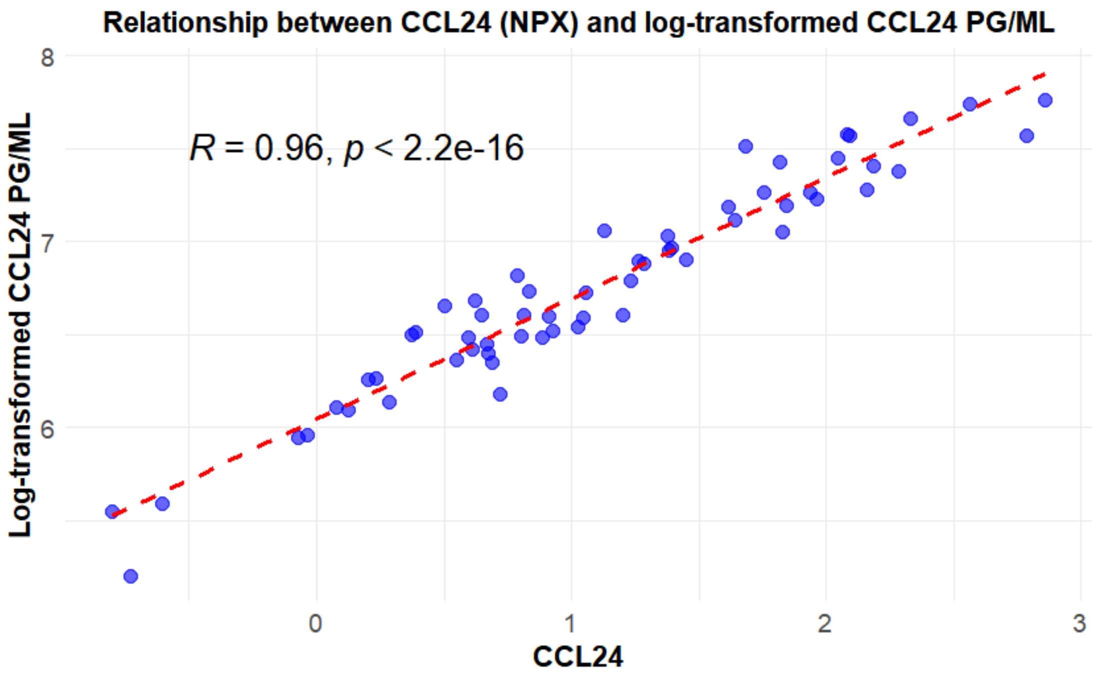


Correlation between NPX levels of CCL24, measured by Olink PEA and serum levels of CCL24 measured by ELISA

Supp. Figure S3

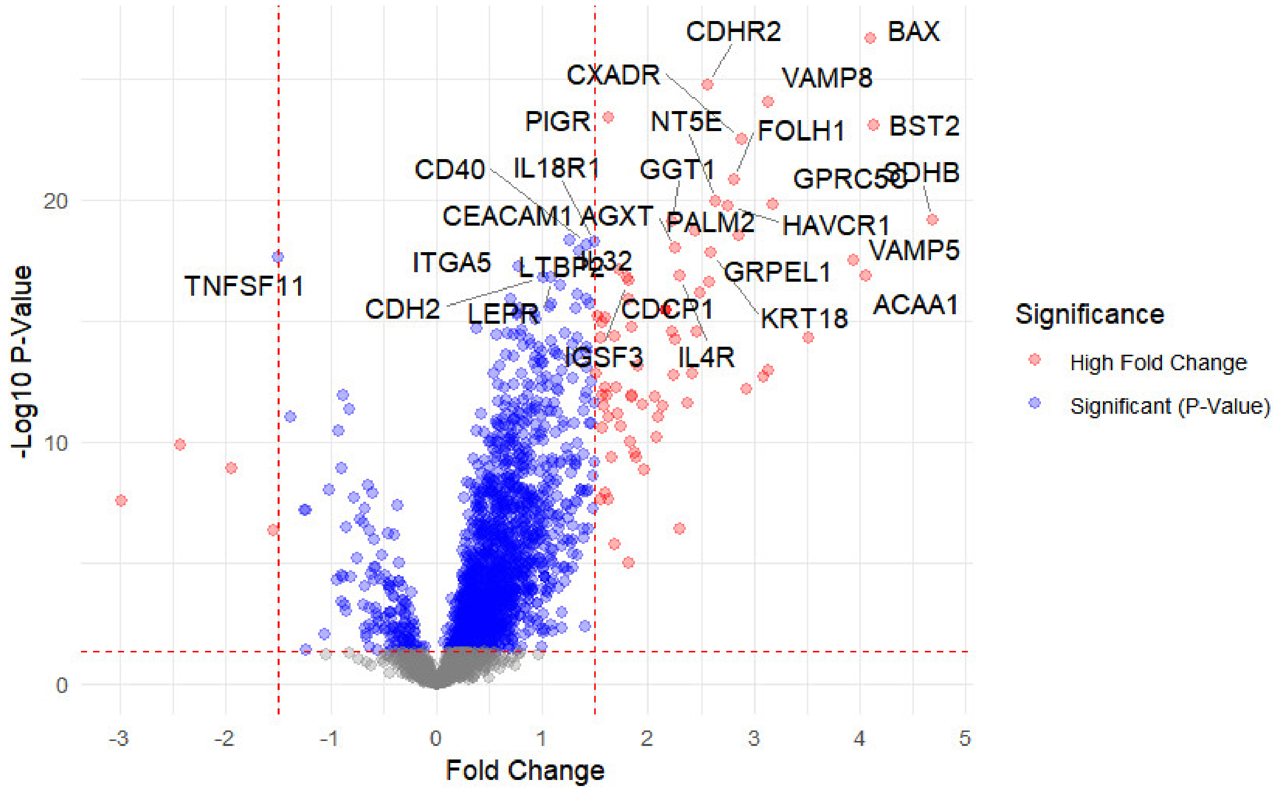


Annotated Volcano Plot of the 30 strongest differentially expressed proteins between healthy controls and patients with PSC.
